# Metformin Treatment Suppresses Melanoma Cell Growth and Motility through Modulation of microRNA Expression

**DOI:** 10.3390/cancers11020209

**Published:** 2019-02-11

**Authors:** Hui-Wen Tseng, Sung-Chou Li, Kuo-Wang Tsai

**Affiliations:** 1 Department of Dermatology, Kaohsiung Veterans General Hospital, Kaohsiung 81362, Taiwan; hwtseng@vghks.gov.tw; 2 Institute of Biomedical Sciences, National Sun Yat-Sen University, Kaohsiung 80424, Taiwan; 3 Genomics & Proteomics Core Laboratory, Department of Medical Research, Kaohsiung Chang Gung Memorial Hospital and Chang Gung University College of Medicine, Kaohsiung 83301, Taiwan; raymond.pinus@gmail.com; 4 Department of Medical Education and Research, Kaohsiung Veterans General Hospital, Kaohsiung 81362, Taiwan; 5 Department of Chemical Biology, National Pingtung University of Education, Pingtung 90004, Taiwan

**Keywords:** metformin, miR-192-5p, miR-584-3p, migration, melanoma

## Abstract

Melanoma is a highly aggressive cancer with high mortality in advanced stages. Metformin is an oral biguanide drug used for diabetes and has demonstrated positive effects on cancer prevention and treatment. Herein, we found that metformin significantly suppressed melanoma cancer cell motility and growth through inducing cell cycle arrest at the G2/M phase and promoting cell apoptosis. Using the next-generation sequencing approach, we identified three upregulated microRNAs (miRNA; miR-192-5p, miR-584-3p, and miR-1246) in melanoma cells treated with metformin. Among these, we examined the roles of miR-192-5p and miR-584-3p and discovered that they significantly suppressed melanoma cell motility. Furthermore, they inhibited melanoma cell growth through destroying cell cycle progression and inducing cell apoptosis. Using microarray and bioinformatics approaches for identifying putative target genes, Epidermal growth factor (EGF) containing fibulin-like extracellular matrix protein 1 (EFEMP1) gene for miR-192-5p and an isoform of the secretory carrier membrane proteins (SCAMP3) gene for miR-584-3p could be silenced through targeting their 3′UTR region directly. EFEMP1 and SCAMP3 knockdown significantly suppressed melanoma cell growth, but only EFEMP1 knockdown inhibited its motility abilities. Our findings indicated that miR-192-5p and miR-584-3p might contribute to metformin-induced growth and motility suppression in melanoma cells through silencing their target genes EFEMP1 and SCAMP3.

## 1. Introduction

The worldwide incidence of melanoma has been increasing continuously for more than 30 years. Melanoma is considered the most aggressive form of human skin cancer with easily metastasis. A patient with melanoma has a considerably low five-year survival rate [1]. Tumorigenesis of melanoma is a multistep process that involves the accumulation of multiple mutations, occurrence of epigenetic alterations, aberration of the transcriptome, and transformation of healthy melanocytes into melanoma cells [2].

MicroRNAs (miRNAs) are small endogenous nonprotein coding RNA molecules that anneal to the 3′ untranslated region (3′UTR) of targeted mRNA molecules through short complementary “seed sequences” [3]. This interaction results in mRNA cleavage or repression of productive translation, thereby silencing the expression of the targeted gene [3]. A particular miRNA can bind to hundreds of various mRNAs, and these miRNAs are estimated to target more than 30% of all protein coding genes in the human genome [4,5]. Some studies have demonstrated that miRNAs, including miR-15b, miR-204, miR-331, miR-342, miR-367, miR-622, miR-612, and let-7b, play key roles in melanoma cancer progression [6,7,8,9,10,11,12,13].

Metformin is an oral antihyperglycemic agent that improves glucose tolerance in patients with type 2 diabetes mellitus by reducing both basal and postprandial plasma glucose levels. Emerging epidemiological evidence has suggested that metformin can be useful in the prevention and treatment of cancer; for example, it may reduce the risk of colorectal, pancreatic, hepatocellular, breast, and lung cancers [14,15,16,17,18]. Some studies have reported that metformin suppresses cancer cell growth by altering miRNA expression in human cancers, namely breast cancer, renal cell carcinoma, cholangiocarcinoma, and pancreatic cancer [19,20,21,22]. Cerezo et al. documented that metformin blocked melanoma cell invasion and metastasis by modulating 5′ adenosine monophosphate-activated protein kinase (AMPK)/p53 signalling [23]. Li et al. reported that metformin could attenuate melanoma cell growth and metastasis through suppressing Tribbles homolog 3 (TRIB3) expression and Lysine acetyltransferase 8 (KAT8)-mediated Spinal muscular atrophy (SMA)-and Mothers decapentaplegic (MAD)-related protein 3 (SMAD3) acetylation [24]. However, the underlying anticancer mechanism of metformin on the regulation of miRNAs in melanoma remains unclear [25]. In this study, we used the next-generation sequencing (NGS) approach to perform small RNA profiling to identify metformin-regulating miRNAs. Furthermore, we explored the effects of miRNAs on anti-melanoma cell growth and motility of metformin.

## 2. Results

### 2.1. In Vitro Metformin Treatment Suppressed Melanoma Cell Growth and Motility

**Figure 1:**
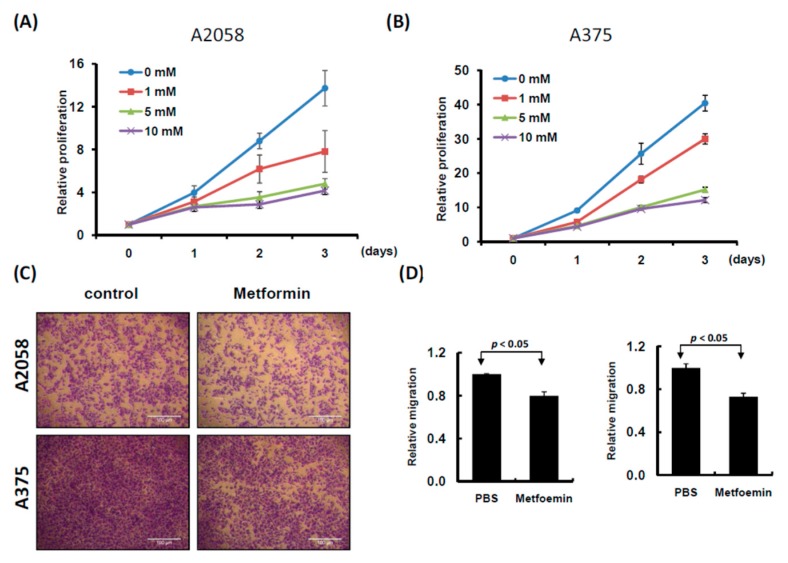
Proliferation and motility of melanoma cells were suppressed with metformin treatment. (**A**) A2058 cells and (**B**) A375 cells were treated with 0, 1, 5 and 10 mM metformin for three consecutive days. Cell growth was examined using CellTiter-Glo One solution assay. (**C**) and (**D**) Cell migration assay was used to examine A2058 and A375 cells treated with or without metformin (5 mM) for three days (magnification, 100×). Migrating cells were stained with crystal violet solution, and the migration abilities were quantified through measuring three different fields under a phase-contrast microscope.

**Figure 2:**
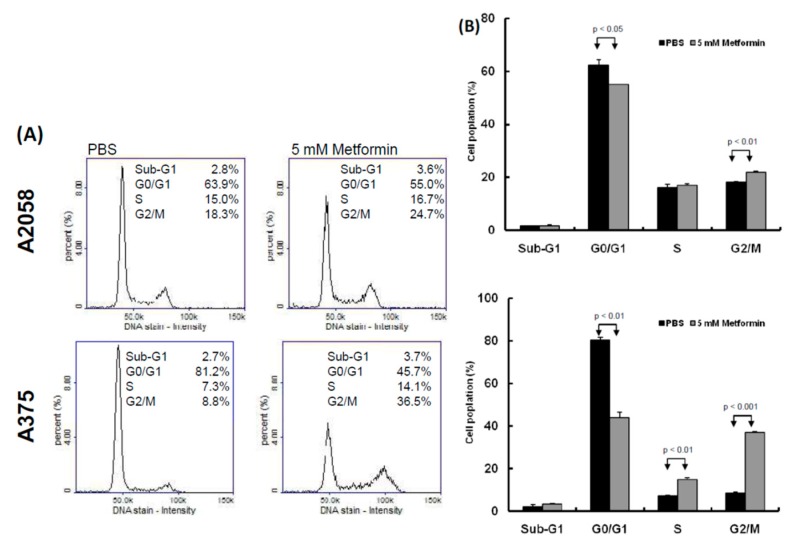

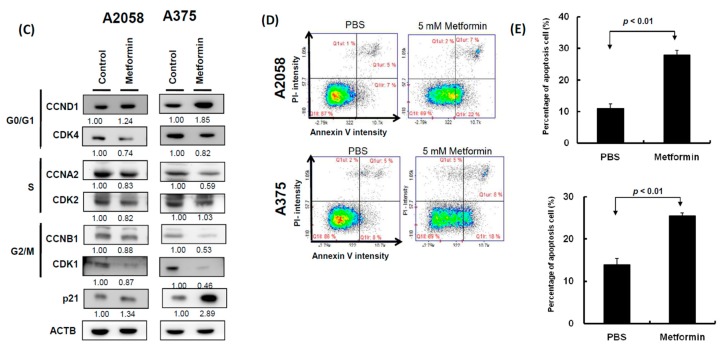
Metformin suppressed melanoma cell growth by impairing cell cycle progression and inducing apoptosis. (**A**) and (**B**) After treatment with 5 mM metformin for three days, cell cycles of A2058 and A375 cells were examined using image-flow cytometry assay**,** and the graph depicts the quantification for each phase. (**C**) Expression levels of cell cycle-relative genes were examined in A2058 and A375 cells treated with or without metformin using the Western blot approach. (**D**) A2058 and A375 cells were treated with or without metformin (5 mM) for three days and stained with propidium iodide; then, cell apoptosis was assessed through image-flow cytometry analysis. (**E**) Percentages of apoptotic cells of A2058 and A375 cells with or without metformin treatment are presented. Data are presented as the mean ± standard deviation of three independent experiments.

Two human melanoma cell lines, A2058 and A375, were treated with various concentrations of metformin (0, 1, 5, and 10 mM) for three consecutive days. Cell proliferation was examined using the CellTiter-Glo One solution assay at day 0, 1, 2, and 3. As depicted in Figure 1A,B, the growth of more than 70% of cells was significantly suppressed on day 3 after treatment with 5 or 10 mM metformin compared with no metformin treatment. Furthermore, the suppressive effects of 0, 1, and 5 mM metformin were dose-dependent (Figure 1A,B); however, the suppressive effects of 5 and 10 mM metformin were similar. On the basis of these results, we used 5 mM metformin to treat melanoma cells for the following assays. According to the results of the migration assay, the migration ability of A2058 and A375 cells was significantly suppressed after treatment with 5 mM metformin (Figure 1C,D). Similar results were also observed in the MeWo and RPMI7951 but not in Hs695T cells (Figure S1). Furthermore, the effects of metformin-induced inhibition of motility were observed only in the MeWo cells, but not in the RPMI7951 and Hs695T cells (Figure S1). We further investigated the effects of metformin on the cell cycle of melanoma cells by using image-flow cytometry analysis. When A2058 and A375 cells were incubated with 5 mM metformin, the number of cells at the S and G2/M phases increased and the number of cells at the G0/G1 phase decreased compared with those in the control group (Figure 2A,B). Similarly, metformin could significantly suppress the growth of MeWo and RPMI7951 cells by impairing cell cycle progression, whereas no effect was observed on the Hs695T cells (Figure S1). Furthermore, the expression levels of CCNA2, CCNB1, CDK1, and CDK4 decreased in melanoma cells treated with metformin, whereas the levels of p21 and CCND1 increased (Figure 2C). In addition, the population of apoptotic cells significantly increased in A2058 and A375 cell lines after 5 mM metformin treatment (Figure 2D,E). These results indicated that melanoma cells growth could be suppressed after metformin treatment through impairing cell cycle progression and inducing cell apoptosis.

### 2.2. Identifying Metformin Treatment-Regulated miRNAs in Melanoma Cells through the NGS Approach

**Figure 3:**
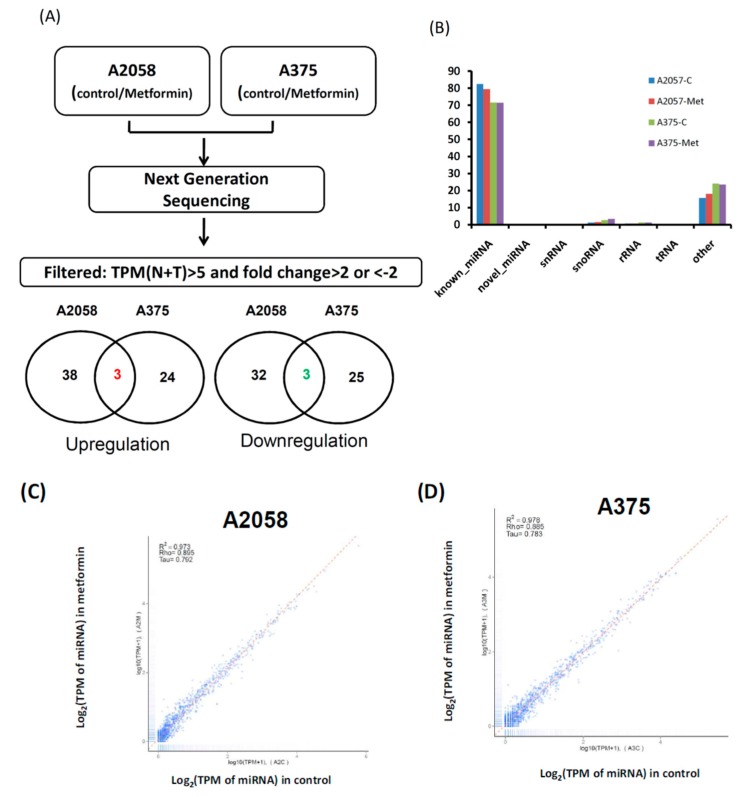
Flowchart of process to identify metformin-regulated miRNA candidates in melanoma cells through next generation sequences (NGS). (**A**) Small profiles of A2058 and A375 cells treated with or without metformin were analyzed using NGS. Comparison of the miRNA expression profiles between the control and metformin treatment samples and filtering steps are as follows: (1) fold change ≥ 2 or ≤ 2 and (2) sums of Transcript per million (TPM) in control and treatment groups ≥ 5. Venn diagrams depict the number of upregulated and downregulated miRNA candidates in A2058 and A375 cells treated with or without metformin. (**B**) Categories of sequence reads in four libraries were detected after mapping the human database. (**C**) Scatter plot of miRNA distribution in A2058; metformin treatment (*Y*-axis) vs. control group (*X*-axis); (**D**) scatter plot of miRNA distribution in A375; metformin treatment (*Y*-axis) vs. control group (*X*-axis).

Our data indicated that 5 mM metformin treatment significantly inhibited the growth and motility of melanoma cells. To explore the role of miRNAs in metformin-suppressing cell growth and motility, small transcriptome profiles of A2058 and A375 cells treated with and without 5 mM metformin were analyzed using the NGS approach (Figure 3A). More than 11 million clean reads in four libraries were identified through the NGS analysis (Table S1). After mapping to the human database (miRBase 19), more than 70% of the clean reads were defined as miRNAs (Figure 3B) and approximately 1100 types of known miRNAs were detected at a level of at least 1 TPM (>1) in each individual library (Table S1). As shown in Figure 3C and Figure 1D, the expression levels were almost consistent in A2058 and A375 cells treated with and without metformin (A2058 *R*^2^ = 0.973 and A375 *R*^2^ = 0.978), implying that only a small fraction of these miRNAs were expressed as more than a twofold change (fold change ≥ 2 or ≤ 2) between melanoma cells treated with and without metformin. A Venn diagram (Figure 3A below panels) depicts the number of miRNAs with differential expression, revealing that 41 types of miRNAs showed increased expression and 35 types of miRNAs showed decreased expression in A2058 cells after metformin treatment. Furthermore, 27 types of miRNAs were up-regulated and 28 types of miRNAs were down-regulated in A375 cells treated with metformin (Figure 3A, below panels). Among them, three types of miRNAs, namely miR-192-5p, miR-584-3p, and miR-1246, showed increased expression (fold change ≥ 2), whereas three types of miRNAs, namely miR-199b-5p, miR-573, and miR-7974, showed decreased expression (fold change ≤ 2) in both A2058 and A375 cells after treatment with 5 mM metformin. Because metformin suppressed the growth of melanoma cells, it should have upregulated tumor-suppressive miRNAs and downregulated oncogenic miRNAs. A review of other studies verified that miR-192-5p and miR-584-3p act to suppress tumors in human cancer [26,27]. Therefore, we selected miR-192-5p and miR-584-3p as our study objects.

### 2.3. miR-192-5p and miR-584-3p Suppressed Melanoma Cell Growth

**Figure 4:**
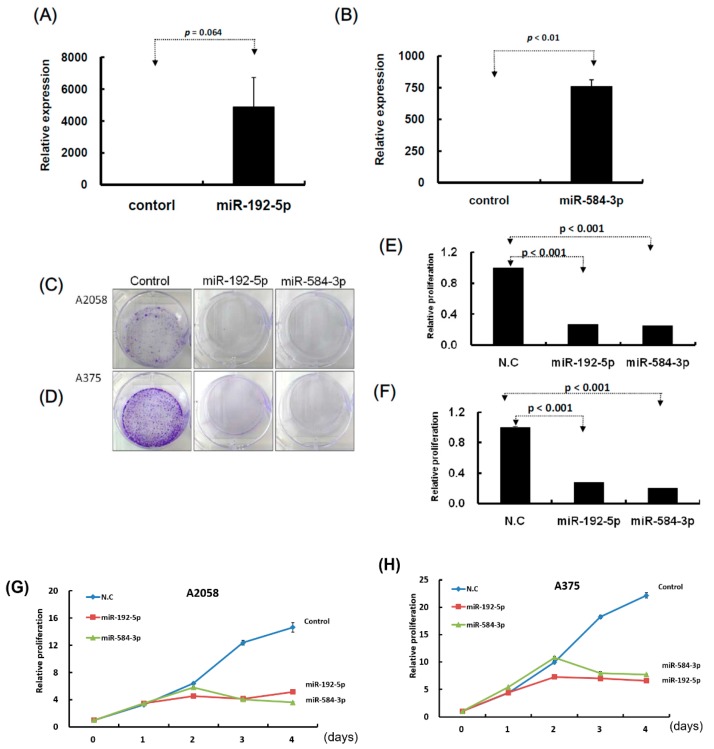
Growth of melanoma cells was suppressed after transfection of miR-192-5p and miR-584-3p mimics candidates. (**A**) and (**B**): After transfection of miRNA mimics, the relative expression levels of miR-192-5p and miR-584-3p were examined through real-time PCR. (**C**) and (**D**): After miR-192-5p, miR-584-3p, and control, respectively, were transfected into melanoma cells, the cells were incubated for two weeks and the colony was examined with crystal violet. (**E**) and (**F**): Relative colony formation ability was quantified using OD595 nm. (**G**) and (**H**): Proliferation of melanoma cells with or without miR-192-5p or miR-584-3p overexpression was examined on four consecutive days using the CellTiter-Glo One solution assay.

**Figure 5:**
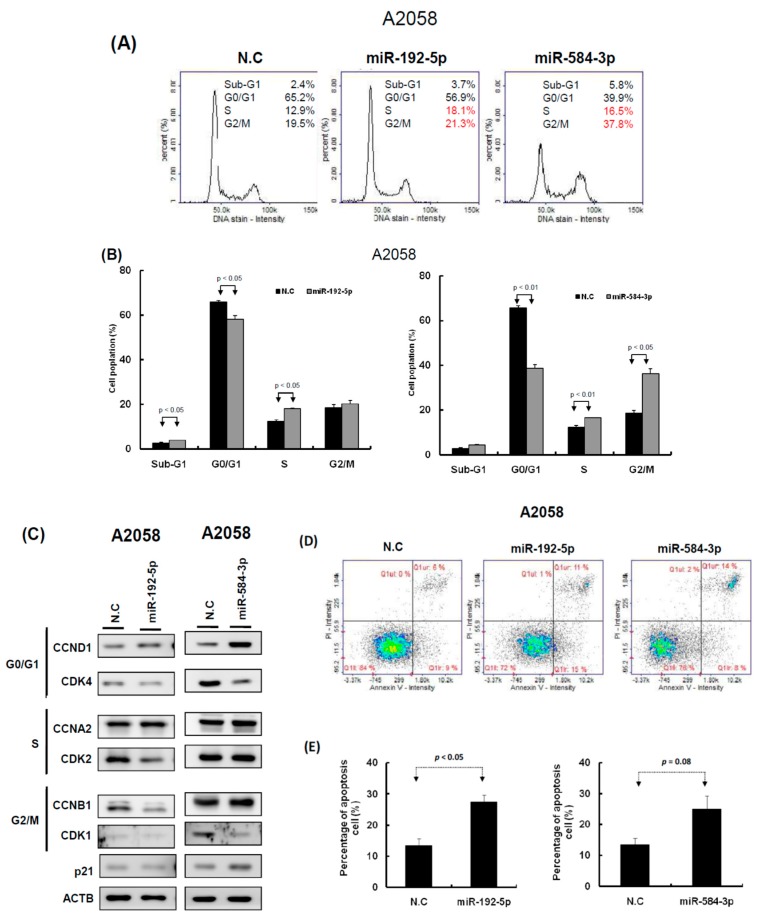
miR-192-5p and miR-584-3p impaired the cell cycle progression of melanoma cells. (**A**) After miR-192-5p or miR-584-3p transfection for three days, the cell cycle distributions of A2058 were examined by image-flow cytometry analysis. (**B**) miR-192-5p and miR-584-3p increased the number of cells in the G2/M and S phase and reduced the number of cells in the G0/G1 phase compared with the control cells. (**C**) Expression levels of cell cycle-relative genes were examined in A2058 cells with miR-192-5p, miR-584-3p, and scramble control transfection using Western blot approach. (**D**) After A2058 cells were transfected with miR-192-5p, miR-584-3p, or scramble control for three days, cells were stained with propidium iodide, then cell apoptosis was assessed by image-flow cytometric analysis. (**E**) Percentage of apoptotic cells quantified. Data are presented as the mean ± standard deviation of three independent experiments.

**Figure 6:**
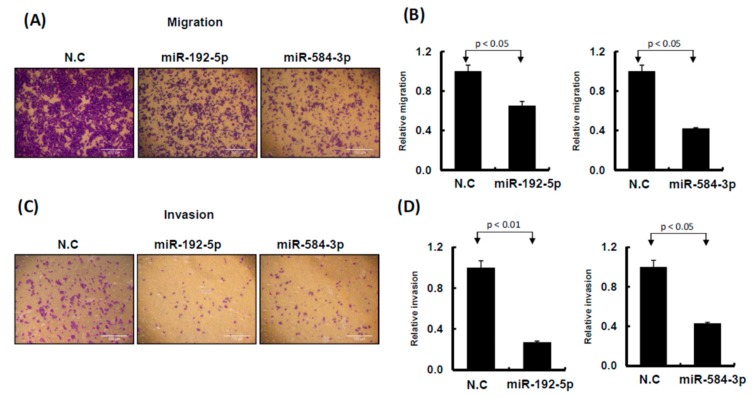
miR-192-5p and miR-584-3p influenced cell invasion ability of melanoma cells. (**A**) and (**C**): Migration and invasion ability were examined by Transwell assay in A2058 cells. After miR-192-5p, miR-584-3p mimics, or scramble control transfection for 24 h, cells were subjected to Transwell assay. Migrating and invading cells were stained with crystal violet solution. (**B**) and (**D**): Numbers of migrating or invading cells were quantified by counting three different fields under a phase-contrast microscope. The cell photographs from a representative experiment are depicted and the graph data were quantified using Ascent software. Data are reported as colonies compared with control (means ± SD).

To understand the functional roles of these metformin-regulated miRNAs in melanoma cell lines, we performed the gain-of-function assay by using the miRNA mimics transfection approach. The expression levels of individual miRNA in A2058 cells after miRNA mimics transfection were confirmed through real-time PCR. The expression levels of miR-192-5p and miR-584-3p in A2058 cells were significantly increased after transfection with mimics of miR-192-5p or miR-584-3p, respectively (Figure 4A,B). Furthermore, we investigated the colony formation ability of melanoma cells after miR-192-5p and miR-584-3p overexpression. The colony formation ability of melanoma cells was clearly suppressed after transfection with miR-192-5p and miR-584-3p mimics (Figure 4C–F). Similarly, the results of the cell proliferation assay revealed that cell proliferation was suppressed by miR-192-5p and miR-584-3p in melanoma cells (Figure 4G,H). Further examination of the cell cycle distribution indicated that the cell cycle was significantly arrested at S and G2/M after ectopic expression of miR-192-5p and miR-584-3p (Figure 5A,B). As depicted in Figure 5C, the expression levels of CDK4 and CDK1 were consistently decreased, whereas those of CCND1 increased in A2058 cells after transfection with miR-192-5p mimics and miR-584-3p mimics. In addition, the population of apoptotic cells significantly increased when miR-192-5p or miR-584-3p was overexpressed in A2058 cells (Figure 5D,E). We also assessed the effects of transfection with miR-192-5p and miR-584-3p mimics on the cell invasion ability of A2058 and A375 melanoma cells. The invasion ability of A2058 cells was clearly suppressed by miR-192-5p and miR-584-3p (Figure 6A–D). Similar results revealed that miR-192-5p and miR-584-3p could significantly suppress the growth and motility of the MeWo, RPMI7951, and Hs695T cells (Figures S2 and S3). Furthermore, miR-192-5p and miR-584-3p also could impair cell cycle progression (Figure S2). To summarize, miR-192-5p and miR-584-3p affected the invasion ability of melanoma cells and suppressed their growth by inducing cell cycle arrest at the S and G2/M phase and triggering the progression of cell apoptosis. To confirm the role of miR-192-5p and miR-584-3p in metformin-induced inhibition of motility, we attempted to improve the effects of metformin-induced inhibition of motility ability of melanoma cells by using miR-192-5p and miR-584-3p inhibitor transfection. As indicated in Figure S4, the metformin-induced motility ability repression in melanoma cells could be partially improved after miR-192-5p and miR-584-3p inhibitor transfection.

### 2.4. Identification of the Putative Target Genes of miR-192-5p and miR-584-3p by Using the Microarray Approach

**Figure 7:**
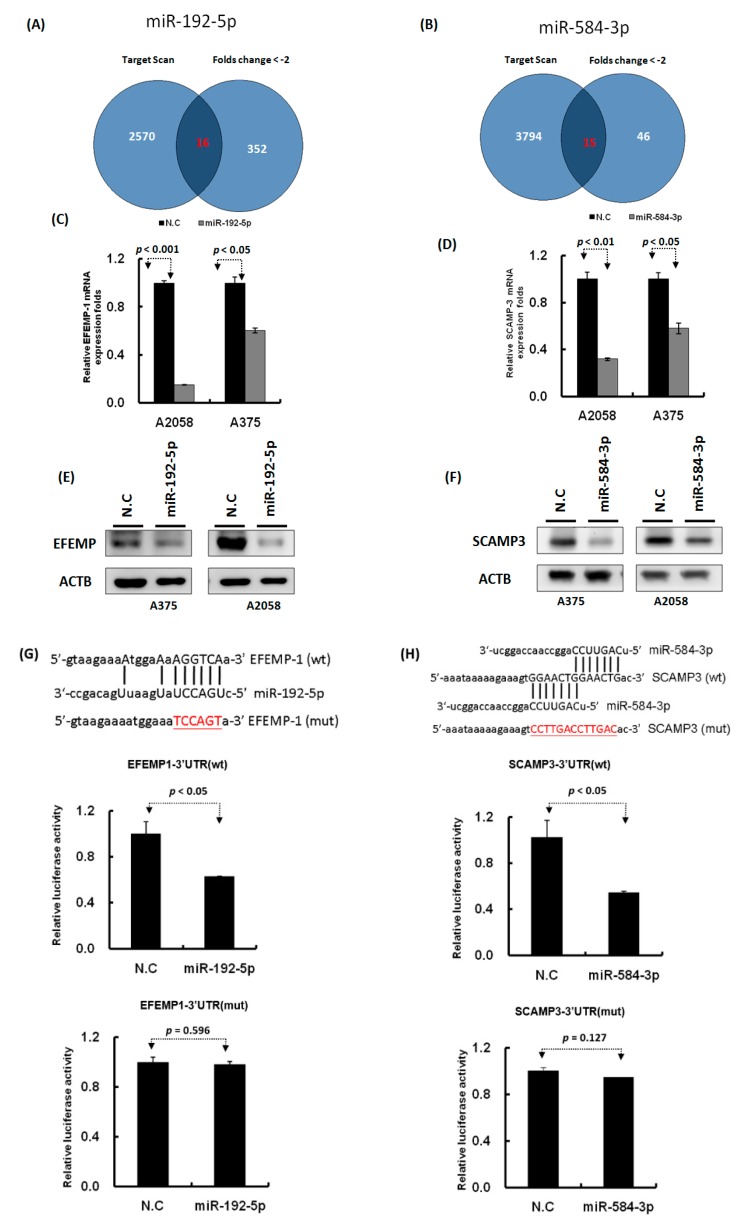
Identification of the putative targets of miR-192-5p and miR-584-3p through microarray and bioinformatics approaches. (**A**) and (**B**): Venn diagrams indicating the numbers of target genes of miR-192-5p and miR-584-3p that were identified using the TargetScan tool and the microarray approach. (**C**) and (**D**): Expression levels of EFEMP1 and SCAMP3 were examined through real-time PCR in melanoma cells with miR-192-5p and miR-584-3p transfection. (**E**) and (**F**): Expression levels of EFEMP1 and SCAMP3 were examined through Western blotting in melanoma cells with miR-192-5p and miR-584-3p transfection. (**G**) and (**H**): Schema of the luciferase constructs (upper panel). The miR-192-5p or miR-584-3p target sequence in the 3′UTR region of their target genes are depicted in the upper panels and the mutant of its 3′UTR was illustrated in red. Relative luciferase activity of the reporter with the wild-type 3′UTR (middle panels) and mutant 3′UTR (lower panels) of EFEMP1 and SCAMP3 genes was determined after co-transfection with miR-192-5p or miR-584-3p mimics in A2058 cells. Firefly luciferase activity served as a transfection control.

To identify the target genes of miR-192-5p and miR-584-3p, we performed three RNA transcriptome profiling of A2058 cells with miR-192-5p mimics, miR-584-3p mimics, and scramble control transfection. We identified 368 protein coding genes that were significantly decreased (fold change ≤ 2), *p* < 0.05) in the A2058 cell line after transfection with miR-192-5p mimics for 48 h. In addition, the TargetScan prediction tool revealed that miR-192-5p could regulate 2586 types of genes through directly targeting their 3′UTR region. Combining these two sets of data, we discovered 16 types of genes that were the possible target genes of miR-192-5p in the A2058 cell line (Figure 7A and Table S2). Using the same criteria, 15 putative genes were identified for miR-584-3p. Among these, we selected three targets for miR-192b-5p (EFEMP1, CTH, and RTL4) and three targets for miR-584-3p (SCAMP3, PSMB1, and TM4SF19); their expression levels were examined with real-time PCR in A2058 and A375 cells with miR-192-5p and miR-584-3p mimic transfection, respectively. EFEMP1 expression could be suppressed in both A2058 and A375 cells with miR-192-5p transfection, and the expression of SCAMP3 and TM4SF19 also could be silenced in A2058 and A375 cells with miR-584-3p overexpression (Figure 7C,D and Figure S5). Our resulted revealed that both miR-192-5p and miR-584-3p played a tumor-suppressive role in the growth and migration of melanoma cells; therefore, their targets should be oncogenes. According to aforementioned results, we selected EFEMP1 and SCAMP3 for further examination. The results of Western blotting assay (Figure 7E,F) indicated that protein levels of EFEMP1 and SCAMP3 were also significantly decreased after transfection with miR-192-5p and miR-584-3p mimics, respectively.

We further constructed the wild-type and mutant 3′UTR region of EFEMP1 and SCAMP3 into the pmiR-reporter vector (Figure 7G,H). The luciferase activity of wild-type EFEMP1-3′UTR significantly decreased (*p* < 0.05) in the A2058 cell line transfected with miR-192-5p mimics, as determined through the luciferase reporter assay (Figure 7G middle panel), whereas the luciferase activity of mutant EFEMP1-3′UTR for miR-192-5p’s binding site was not altered (Figure 7G lower panel). Using the same approach, we determined that the luciferase activity of wild-type SCAMP3-3′UTR significantly decreased (*p* < 0.05) in the A2058 cell line transfected with miR-584-3p mimics (Figure 7H middle panel); however, the luciferase activity of mutant SCAMP3-3′UTR was unchanged (Figure 7H lower panel). These results indicated that miR-192-5p could inhibit EFEMP1 expression and miR-584-3p could suppress SCAMP3 expression by directly targeting their 3′UTR regions.

### 2.5. Knockdown of EFEMP1 and SCAMP3 Suppressed Melanoma Cell Growth

**Figure 8:**
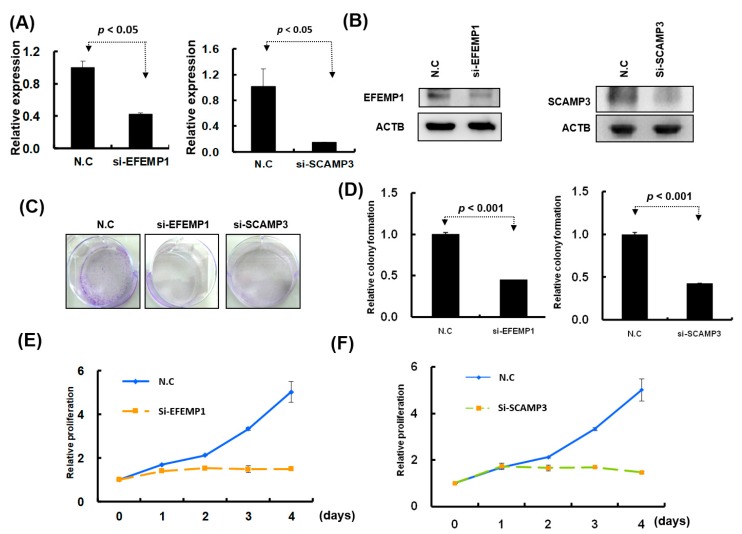
Knockdown of EFEMP1 and SCAMP3 expression significantly inhibited melanoma cell growth. (**A**) and (**B**): After siRNA transfection, the expression levels of EFEMP1and SCAMP3 were examined through real-time PCR and Western blotting. (**C**) and (**D**): Colony formation assay was examined in A2058 cells with EFEMP1 and SCAMP3 knockdown; and the relative colony formation ability was quantified using OD595 nm. (**E**) and (**F**): Cell proliferation was assessed in A2058 cells with EFEMP1 and SCAMP3 knockdown using the CellTiter-Glo One solution assay.

**Figure 9:**
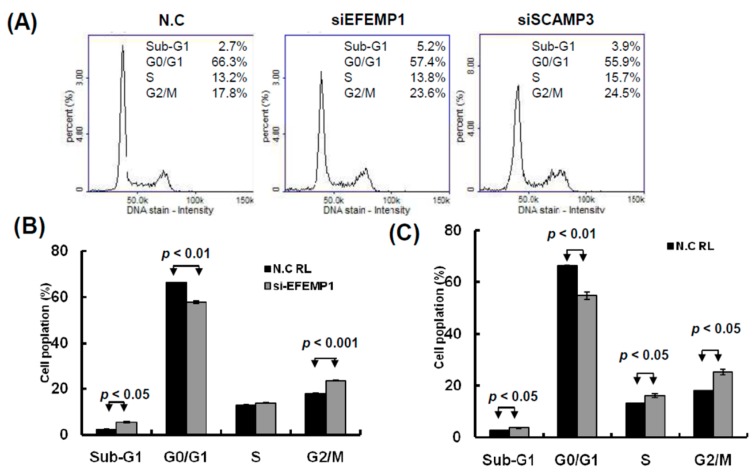
Knockdown of EFEMP1 and SCAMP3 impaired the cell cycle progression of melanoma cells. (**A**) After siEFEMP1, siSCAPM3, or scramble control transfection for three days, the cell cycle distributions of A2058 were examined by image-flow cytometry analysis. (**B**) EFEMP1 and SCAMP3 knockdown increased the number of cells in the G2/M and S phase and reduce the number of cells in the G0/G1 phase compared with the control cells. (**C**) After A2058 cells were transfected with siEFEMP1, siSCAPM3, or scramble control for three days, cells were stained with propidium iodide, then cell apoptosis was assessed by image-flow cytometric analysis. (**D**) Percentage of apoptotic cells was quantified and was presented. Data are presented as the mean ± standard deviation of three independent experiments.

**Figure 10:**
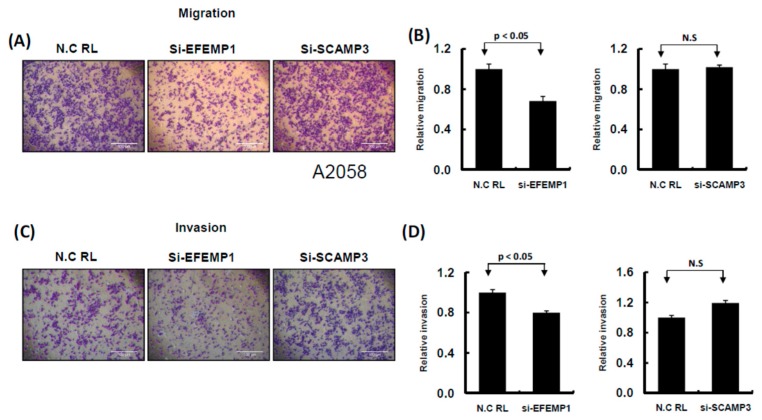
Knockdown of EFEMP1 and SCAMP3 expression influenced the cell invasion ability of melanoma cells. (**A**) and (**C**): Migration and invasion ability were examined by Transwell assay in A2058 cells. After knockdown of EFEMP1 and SCAMP3, or scramble control transfection for 24 h, cells were subjected to Transwell assay. The migrating and invading cells were stained with crystal violet solution. (**B**) and (**D**): Then, the numbers of migrating or invading cells were quantified by counting three different fields under a phase-contrast microscope. The cell photographs from a representative experiment are presented and the graph data were quantified using Ascent software. Data are reported as colonies compared with control (means ± SD).

**Figure 11:**
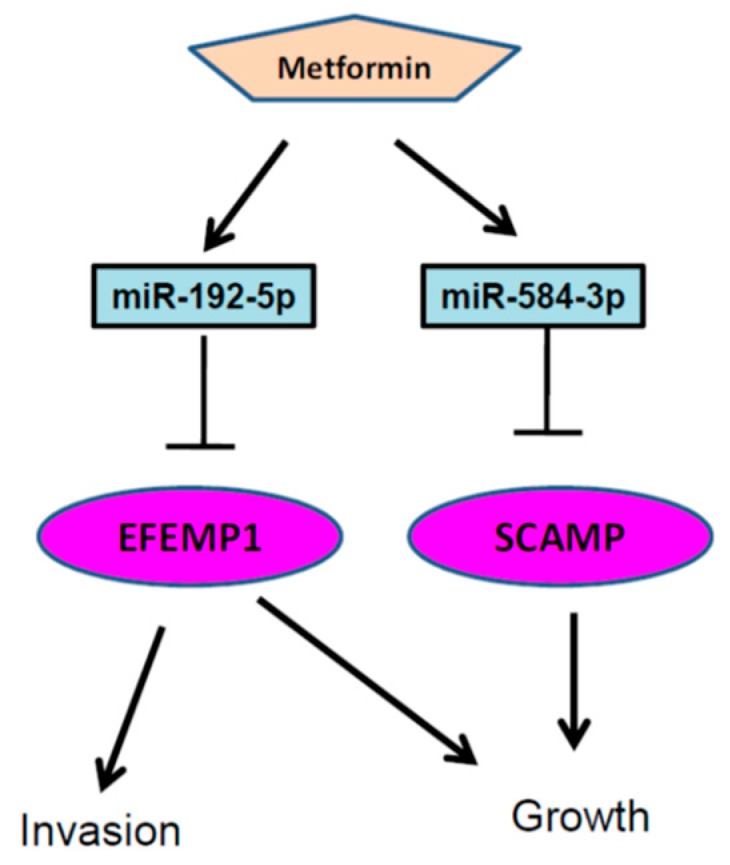
Putative model describing that metformin suppressed melanoma cell growth and motility through modulating the miR-192-5p/EFEMP1 and miR-584-3p/SCAMP3 axes.

To understand the functions of EFEMP1 and SCAMP3, we performed a loss-of-function assay by using the siRNA transfection approach. After transfection of si-EFEMP1 and si-SCAMP3 into melanoma cells, the expression levels of individual genes were confirmed through Western blotting and real-time PCR. The expression levels of EFEMP1 and SCAMP3 were significantly lower than that of the scramble control in A2058 cells transfected with si-EFEMP1, si-SCAMP3, or scramble control (Figure 8A,B). We further investigated the effects of EFEMP1 and SCAMP3 knockdown on cell growth. Cell colony formation and proliferation were considerably suppressed by EFEMP1 and SCAMP3 knockdown (Figure 8C–E). In addition, EFEMP1 and SCAMP3 knockdown substantially induced cell cycle arrest at G2/M and increased the sub-G1 population in A2058 cells (Figure 9A–C), respectively. As depicted in Figure 10A–D, cell invasion and migration were clearly suppressed by EFEMP1 and were not changed by SCAMP3 knockdown. The results indicated that metformin treatment suppressed the motility and growth of melanoma cells because it might directly modulate miR-192-5p-EFEMP1 and miR-584-3p-SCAMP3 axes in melanoma cells (Figure 11).

## 3. Discussion

Metformin has been widely used as the first choice of oral hypoglycemic agent for type 2 diabetes for decades. Some cell lines, animal models, and clinical studies have proposed that metformin plays a role in cancer prevention and treatment [16]. Studies have examined the effect of metformin on various cancer types such as pancreatic [28], liver [29], colorectal [16], breast [30] and prostate [31] cancers. A clinical trial study indicated that metformin monotherapy demonstrated no benefits in melanoma patients with an advanced stage of cancer [32]. Other studies have revealed that acidosis-exposed environments could significantly increase the effect of metformin on suppressing melanoma cell growth and motility ability [33,34]. This contradictory result may be attributable to patients with advanced melanoma carrying more gene mutations and highly aggressive melanoma cells that contribute to different microenvironments.

Direct and indirect effects of metformin may work to inhibit cancer. Indirectly, metformin inhibits glucose production in the liver and stimulates glucose uptake in the muscle, thereby enhancing insulin sensitivity and reducing blood glucose and insulin levels. Directly, metformin activates AMPK by inhibiting the complex I of the mitochondrial respiratory chain, which leads to impaired mitochondrial function and conditions that effectively mimic cellular energy stress [35], such as glucose deprivation, hypoxia, oxidative stress, ischemia, and muscle contraction or exercise, all of which lead to the activation of the AMPK axis [36] and inhibit the mammalian target of rapamycin complex 1 (mTORC1).

Drugs operate differently in cell cultures and human physiology, which raises some concerns regarding the applicability of the findings of drug effects in cell cultures to humans. The first concern is that the standard range of the therapeutic plasma level of metformin in humans is 0.465–2.5 mg/L [37], which results when an average patient with a body weight of 60 kg receives a recommended human therapeutic daily dose (1000–2550 mg). Extremely high plasma metformin levels rarely occur in the clinical setting and are only present especially in ill patients with renal dysfunction and lactic acidosis receiving metformin treatment (4–8 mg/L). Reported plasma metformin concentrations have ranged from 0.129 to 90 mg/L, and the lowest and highest boundaries were 0 and 1800 mg/L [38]. The dose of metformin used in this study was 1–10 mM (165–1650 mg/L), leading to 30- to 400-fold excess concentrations that were far in excess of the recommended therapeutic doses. However, the higher concentrations of metformin in vitro studies have usually been used for testing its effect because metformin is typically added once to cell cultures rather than daily, as is done in humans, to maintain a study concentration.

Metformin has been demonstrated to inhibit the growth of cancer cells through inducing G1-phase arrest by reducing the cyclin D1 level in prostate cancer cells [39]. Kato et al. reported that metformin could block the cell cycle in G0–G1 in vitro and in vivo by modulating cell cycle-relative genes in gastric cancer [40]. Wang et al. also reported that metformin inhibited the proliferation of myeloma cells by inducing cell cycle arrest at the G0/G1 phase [41]. Tomic et al. reported that metformin had an antiproliferative effect on melanoma cells, whereas normal human melanocytes were resistant to these metformin-induced effects. Metformin treatment impaired melanoma tumor growth in mice and induced autophagy and apoptosis markers in one study [42]. In our study, we found a similar effect of metformin treatment on melanoma cells: metformin considerably inhibited melanoma cell growth by inducing cell cycle arrest at G2/M and apoptosis. Another study reported that metformin suppressed glioblastoma cell line growth by inducing cell cycle arrest at G2/M [43]. In light of these various results, it appears that metformin can suppress cancer cell growth by impairing cell cycle progression and inducing apoptosis in human cancer cells; however, the effects of metformin on cell cycle progression might vary depending on the cancer type and microenvironments.

In our study, we observed that metformin significantly suppressed melanoma cell motility by modulating the miR-192-5p/EFEMP1 axis. Cerezo et al. demonstrated that metformin inhibited cell invasion through modulating the expression of proteins involved in the epithelial–mesenchymal transition, including Slug, Snail, SPARC, fibronectin, and N-cadherin, and by inhibiting MMP-2 and MMP-9 activation [23]. Furthermore, this process was dependent on the activation of AMPK and the tumor suppressor protein p53. These data reinforced the premise that metformin might be a good candidate for clinical trials of melanoma treatment [23].

Metformin affected miRNA profile expression [44]. Concerning the regulation of miRNA expression by metformin, Li et al. reported that metformin altered the expression profiles of miRNAs, including miR-26a, miR-192, and let-7c, in human pancreatic cancer cells [44]. In the present study, we identified three types of miRNAs—miR-192-5p, miR-584-3p, and miR-1246—that had significantly increased expression levels, whereas miR-199b-5p, miR-573, and miR-7974 had significantly decreased expression levels in melanoma cells treated with metformin. We demonstrated that miR-192-5p and miR-584-3p played crucial roles in the regulation of melanoma cell growth or invasion ability through the targeting of EFEMP1 and SCAMP3, respectively. In other studies, miR-192 has been investigated in vitro and in vivo, and it has been frequently determined to be a tumor suppressor and a potential biomarker in human cancers such as lung cancer [45], colorectal cancer [46], esophageal cancer [47], gastric cancer [48], breast cancer [49], and bladder cancer [50]. Ortega et al. reported that metformin treatment led to a dramatic increase in circulating miR-192 levels [51]. Braun et al. reported that p53-responsive miR-192 and miR-215 were capable of inducing cell cycle arrest [52]. In our study, we also found that miR-192 was up-regulated in melanoma cell lines after metformin treatment and that miR-192 could suppress melanoma cell growth and invasion through directly silencing EFEMP1 expression. One study reported that EFEMP1 was upregulated in malignant gliomas and may play a role in the aggressive nature of these tumors [53]. Yin et al. reported that EFEMP1 promoted ovarian cancer cell growth, invasion, and metastasis through activation of the AKT pathway [54]. In bladder cancer, EFEMP1 was also reported to possess an oncogenic role and to promote bladder cancer metastasis [55]. Similar to these findings, we discovered that EFEMP1 knockdown could significantly suppress melanoma cell growth and invasion ability and induced cell apoptosis; moreover, in the present study, we reported the novel finding that miR-192-5p could significantly suppress melanoma cell growth and invasion ability through directly targeting EFEMP1 expression.

In several studies, miR-584 has been determined to play the role of a tumor suppressor that inhibits cancer cell motility, invasion, and migration through downregulating target oncogene hypoxia-induced Roh-associated protein kinase 1 (ROCK1) in human clear cell renal cell carcinoma [56] and thyroid carcinoma [57], through repressing the transcription of matrix metalloproteinase (MMP)-14 in human neuroblastoma [58], through targeting PTTG1IP in human glioma cells [59], through downregulating WWP1 in human gastric cancer [60], and through directly targeting the MMP-14 promoter to repress YY1-facilitated MMP-14 expression, inhibiting the progression of gastric cancer [27]. miR-584 was also upregulated in melanoma cells after metformin treatment in our study. Furthermore, miR-584 expression significantly inhibited melanoma cell growth through targeting SCAMP3. SCAMP3 is a membrane-trafficking protein involved in endosome transport. Aoh et al. reported that SCAMP3, its modification with ubiquitin, and its interactions with endosomal-sorting complexes required for transport coordinated to regulate endosomal pathways and affected the efficiency of receptor down-regulation. It negatively regulated epidermal growth factor receptor degradation and promoted receptor recycling [61]. Zhang et al. reported that knockdown of SCAMP3 expression suppressed cell proliferation and blocked the cell cycle of hepatocellular carcinoma (HCC) cells [62]. Consistent with these findings, our results indicated that SCAMP3 played an oncogenic role in melanoma cell growth through the regulation of cell cycle progression.

## 4. Material and Methods

### 4.1. Cell Lines and Metformin Treatment

The human melanoma cell lines A375, A2058, HS695T, MeWo and RPMI7951 were cultured in Dulbecco’s modified Eagle’s medium (Invitrogen GIBCO, Carlsbad, CA, USA) supplemented with 10% fetal bovine serum (HyClone, Thermo Scientific, UT, USA), 2 mM glutamine, 10 μg/mL of streptomycin and 100 U/mL of penicillin. Melanoma cells were treated with metformin at various concentrations (1, 5, and 10 mM), and cell growth was observed for three consecutive days.

### 4.2. Extraction of RNA

The total RNA of melanoma cells treated with or without metformin was extracted using TRIzol reagent (Invitrogen, Carlsbad, CA, USA) according to manufacturer’s instructions. The detail process was described in our previous study [63].

### 4.3. Small RNA Library Preparation

In this study, four RNA samples A2058-control (without metformin treatment), A2058-Met (5 mM metformin treatment for three days), A375-control (without metformin treatment), and A375-Met (5 mM metformin treatment for three days)—were sequenced using the MiSeq platform. Briefly, small RNA-sequencing libraries were assembled using the NEBNext small RNA library prep kit (New England Biolabs, Cat#E7300) according to the manufacturer’s protocol. The 3′ and 5′ adaptors were ligated to total RNA, and reverse transcription (RT) was performed; subsequently, polymerase chain reaction (PCR) amplification was performed. PCR products were size-fractionated through 6% polyacrylamide gel electrophoresis, and bands containing 140-nucleotide RNA fragments were selected for purification. Finally, small RNA libraries were sequenced using the Illumina MiSeq platform (150 cycle single read; MiSeq Reagent kit V3_150 cycles; Illumina, San Diego, CA, USA).

### 4.4. Analysis of Small RNA Sequencing Data Using the Bioinformatics Approach

Low-quality reads were removed from the generated sequence reads. Clean reads were obtained through 3′ adaptor trimming. To attain a high confidence level, only clean reads with a read count of ≥2 were included in further analyses. The clean reads were aligned to the miRbase release 19 using bowtie 2 tools. The expression levels of miRNAs in each library were calculated as transcript per million (TPM).

### 4.5. RT and Real-Time PCR of Mature miRNAs

RT primers were designed to align with mature miRNAs for individual miRNA candidates. Complementary DNAs (cDNAs) were obtained from a stem-loop RT reaction by using miRNA-specific stem-loop RT primers and SuperScript III Reverse Transcriptase. This reaction was performed as previously described [64]. Gene expression was detected using an SYBR Green I assay (Applied Biosystems), and expression levels of miRNAs were normalized with U6. The following primers were used:

miR-192-5p-RT: 5′-CTCAACTGGTGTCGTGGAGTCGGCAATTCAGTTGAGGGCTGTCA-3′

miR-192-5p-GSF: 5′-CGGCGGCTGACCTATGAATTGA-3′

miR-584-3p-RT: 5′-CTCAACTGGTGTCGTGGAGTCGGCAATTCAGTTGAGAGCCTGGT-3′

miR-584-3P-GSF: 5′-CGGCGGTCAGTTCCAGGCCAAC-3′

Universal reverse: 5′-CTGGTGTCGTGGAGTCGGCAATTC-3′

U6-F: 5′-CTCGCTTCGGCAGCACA-3′

U6-R: 5′-AACGCTTCACGAATTTGCGT-3′

### 4.6. Ectopic Expression of miRNA Candidates

Melanoma cells were transfected with 10 nM miRNA-192-5p mimics, miR-584-3p mimics, or appropriate miRNA mimics control (GenDiscovery Biotechnology, Inc., Taiwan, using Lipofectamine RNAiMAX reagent, Invitrogen). The expression levels of the mature individual miRNA candidates, miR-192-5p and miR-584-3p, were confirmed through stem-loop qRT-PCR.

### 4.7. Cell Migration and Invasion Assays

Melanoma cells were treated with various concentrations of metformin (5 mM) or transfected with miR-192-5p, miR-584-3p, si-EFEMP1 (epidermal growth factor–containing fibulin-like extracellular matrix protein 1), si-SCAMP3 (secretory carrier membrane protein 3), or scramble control for 48 h. Cell migration and invasion assay was performed as previously described [63].

### 4.8. Cell Proliferation and Colony Formation Assays

For cell proliferation assay, 1 × 10^3^ melanoma cells were seeded in a 96-well plate and treated with various concentrations of metformin (0, 1, 5, and 10 mM) and transfected with miR-192-5p, miR-584-3p, si-EFEMP1, si-SCAMP3, or scramble control. Cell proliferation was assessed on day 0, 1, 2, 3, and 4 by using the CellTiter-Glo One Solution Assay (Promega, Madison, WI, USA). All these experiments were independently repeated three times.

For clonogenic assay, 1 × 10^3^ living cells were deposited onto a 6-well plate and transfected with 10 nM miR-192-5p, miR-584-3p mimics, si-EFEMP1, si-SCAMP3 or scramble control. The detail process was described in our previous study [63].

### 4.9. Image-Flow Cytometry Assay

In this assay, 1 × 10^6^ cells were collected, 70% ethanol was added as the fixative, and cells were incubated at −20 °C overnight. Then, cells were stained with 4′,6-diamidino-2-phenylindole (ChemoMetec, Gydevang, Lillerød, Denmark) and detected using NucleoView NC-3000 software (ChemoMetec, Gydevang, Lillerød, Denmark).

### 4.10. Apoptotic Cells Were Stained with Annexin V, Propidium Iodide, and Hoechst 33342

The apoptotic cells were analyzed by image-flow cytometry assay, and relative process and information was described in our previous study [65].

### 4.11. Knockdown of EFEMP1and SCAMP3 with Small Interfering RNA

Small interfering RNA (siRNA) oligonucleotides targeting EFEMP1 (si-EFEMP1; sense: 5′-GGUGGAAUGGAGUGUGUCATT-3′ and antisense: 5′-UGACACACUUCAUUCCACCTT-3′), SCAMP3 (si-SCAMP3; sense: 5′-CCUAAGAACUAUGGCUCAUTT-3′ and antisense: 5′-AUGAGCCAUAGUUCUUAGGTT-3′), and a scrambled oligo as a negative control were designed and synthesised by GenDiscovery Biotechnology (Taipei, Taiwan). Melanoma cells were transfected with a final concentration (10 nM) of an individual siRNA or control using Lipofectamine RNAiMAX (Invitrogen, Thermo Fisher Scientific). After transfection for 24 h, the protein was extracted and knockdown efficiency was evaluated through Western blotting.

### 4.12. Western Blotting Assay

The western blotting assay was performed as previously described [65]. The following primary antibodies were used in present study: EFEMP1 (GTX111657, GeneTex, Inc, Irvine, CA, USA), SCAMP3 (GTX102216, GeneTex, Inc, Irvine, CA, USA), CCNA2 (1:1000; 18202-1-AP, Proteintech Group, Inc., Rosemont, IL, USA), CCNB1 (1:1000; 55004-1-AP, Proteintech Group, Inc., Rosemont, IL, USA), CCND1 (1:1000; RM-9104-S, Thermo Fisher Scientific Inc., Waltham, MA, USA), p21 (1:1000; #2947, Cell Signaling Technology, Inc., Beverly, MA, USA), CDK1 (1:200; 10762-1-AP, Proteintech Group, Inc., Rosemont, IL, USA), CDK2 (1:100; MS-459-P, Thermo Fisher Scientific Inc., Waltham, MA, USA), CDK4 (1:1000; MS-299-P, Thermo Fisher Scientific Inc., Waltham, MA, USA), actin (MAB1501, Millipore), and GAPDH (GTX627408, GeneTex).

### 4.13. Microarray Analyses

cDNA probes were derived from paired RNA samples obtained from cells transfected with miR-192-5p mimics or a control and cells transfected with miR-584-3p mimics or a control. Microarray experiments and data analyses were performed using the Agilent Oligo Chip manufactured by Welgene Biotech (Taipei, Taiwan). The detail process was described in our previous study [63].

### 4.14. Candidate miRNA Targets and Luciferase Activity Assay

Putative target genes of miR-192-5p and miR-584-3p were predicted using the TargetScan tool (release no. 7.0) [5] and microarray data. In this study, we identified 16 candidate targeting genes for miR-192-5p and 15 candidate genes for miR-584-3p. The sequences and seed region mutants of 3′UTR of EFEMP1 and SCAMP3 were cloned into the pMIR-REPROT vector (AM5795; Thermo Fisher Scientific). Then, pMIR-REPROT-EFEMP1(wt), pMIR-REPROT-SCAMP3(wt), pMIR-REPROT-EFEMP1(mut), or pMIR-REPROT-SCAMP3(mut) was cotransfected with or without miR-192-5p and miR-584-3p mimics into the melanoma cancer cell line by using Lipofectamine 2000 (Invitrogen). After 24 h of transfection, cell lysates were used for measuring luciferase activity by using the Dual-Glo Luciferase Reporter Assay System (Promega, Madison, WI, USA).

## 5. Conclusions

In conclusion, our study demonstrated that metformin treatment suppressed melanoma cell growth and invasion ability, which might be accomplished partially through the modulation of both miR-192-5p/EFEMP1 and miR-584-3p/SCAMP3 axes. Our findings provide new insights into melanoma cancer therapy in the future.

## Supplementary Materials

The following are available online at https://www.mdpi.com/2072-6694/11/2/209/s1, Figure S1: Proliferation and motility of melanoma cells were suppressed after metformin treatment, Figure S2: Growth of melanoma cells was suppressed after transfection of miR-192-5p and miR-584-3p mimic candidates, Figure S3: Motility of melanoma cells was suppressed after transfection with miR-192-5p and miR-584-3p mimic candidates, Figure S4: Partial improvement made by miR-192-5p and miR-584-3p inhibitors in the metformin-induced suppression of melanoma cell motility, Figure S5: Expression levels of CTH, RPL4, PSMB1, and TM4SF19 were examined through real-time PCR in two melanoma cells with miR-192-5p (A and B) and miR-584-3p (C and D) transfection, Table S1: Summary of sequence reads and the detected miRNAs in four libraries, Table S2: Target candidates of miR-192-5p and miR-584-3p were identified using a microarray approach and a bioinformation approach.
