# Supplementary material for: Metformin Treatment Suppresses Melanoma Cell Growth and Motility through Modulation of microRNA Expression
Source: Cancers (Basel). 2019 Feb 11;11(2):209. doi: 10.3390/cancers11020209 (PMC6406940; doi:10.3390/cancers11020209)
Supplement: Supplementary file 1 [file cancers-11-00209-s001.pdf]

cancers

Supplementary Materials: Metformin Treatment Suppresses Melanoma Cell Growth and Motility Through Modulation of microRNA Expression

Hui-Wen Tseng, Sung-Chou Li and Kuo-Wang Tsai

Table S1. Summary of sequence reads and the detected miRNAs in four libraries.

Sample	Total Reads	Clead Reads(%)	miRNAs
**A2058-C**	11909638	11734194 (98.53%)	1155
**A2058-Met**	13141031	12904655 (98.20%)	1261
**A375-C**	12376430	1194227 (96.53%)	1143
**A375-Met**	14184012	13965787 (98.46%)	1229

Table S2. Target candidates of miR-192-5p and miR-584-3p were identified using a microarray approach and a bioinformation approach.

Targets of miR-192-5p	Targets of miR-584-3p
EFEMP1	SCAMP3
CTH	PSMB1
RPL4	TM4SF19
PPP1CA	CABP7
SDS	LPCAT3
KIAA1467	IMP4
ATF3	TYR
PABPC4	BAX
GOT1	GDF15
CSF1	RPL38
PIM1	HLA-DQB2
COPS7A	EI24
PDCD7	DHRS12
SUPT4H1	PPP1R3F
CMTM6	

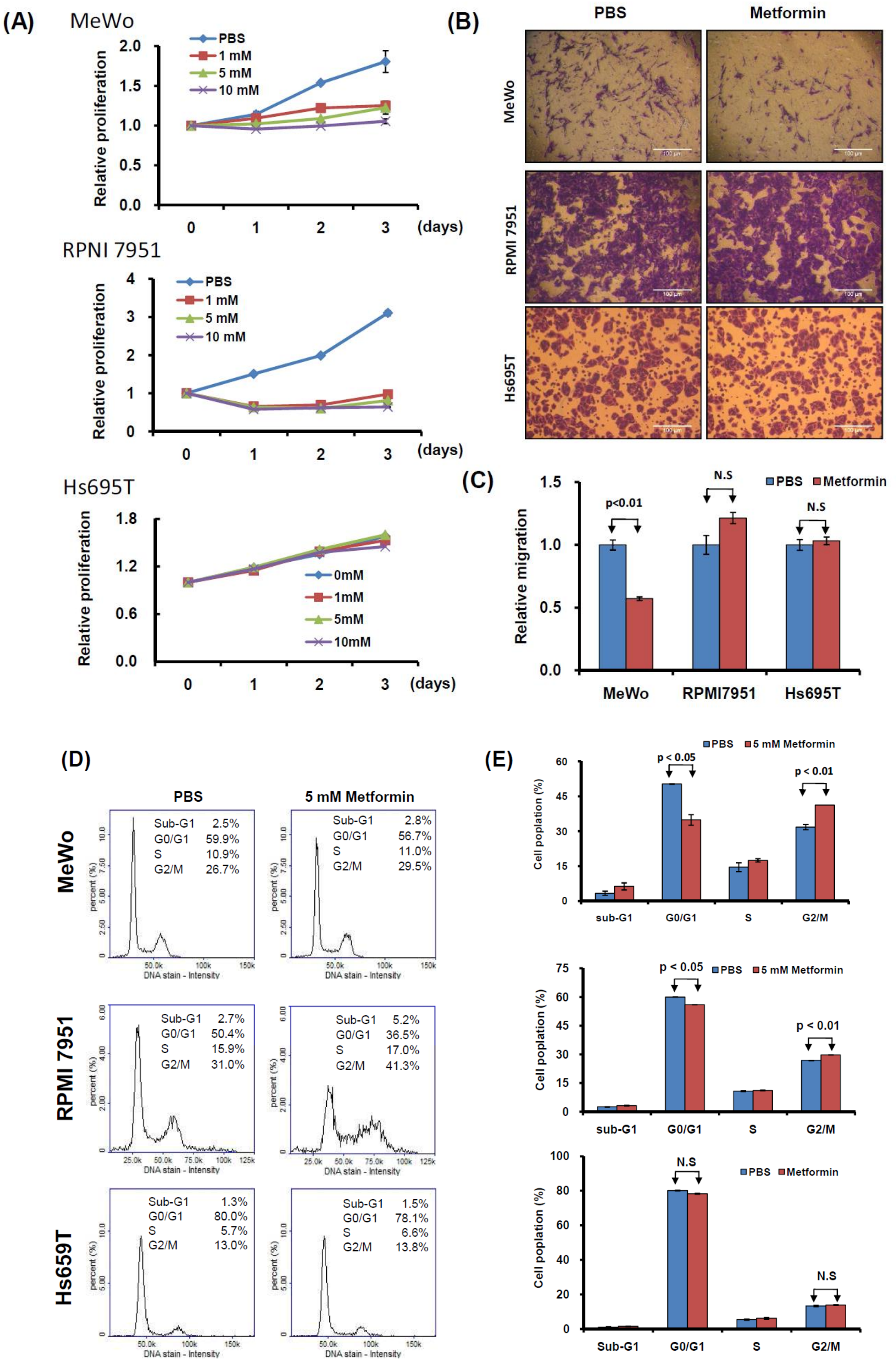

Figure S1. Proliferation and motility of melanoma cells were suppressed after metformin treatment. (**A**) Cell growth was examined in the MeWo, RPMI7951, and Hs695T cells after metformin treatment by using the CellTiter-Glo One solution assay. (**B**) and (**C**) Cell migration assay was used to examine and quantify the MeWo, RPMI7951, and Hs695T cells treated with or without metformin (5 mM) for three days. (**D**) and (**E**) Cell cycle progression was examined and quantified in the MeWo, RPMI7951, and Hs695T cells treated with or without metformin.

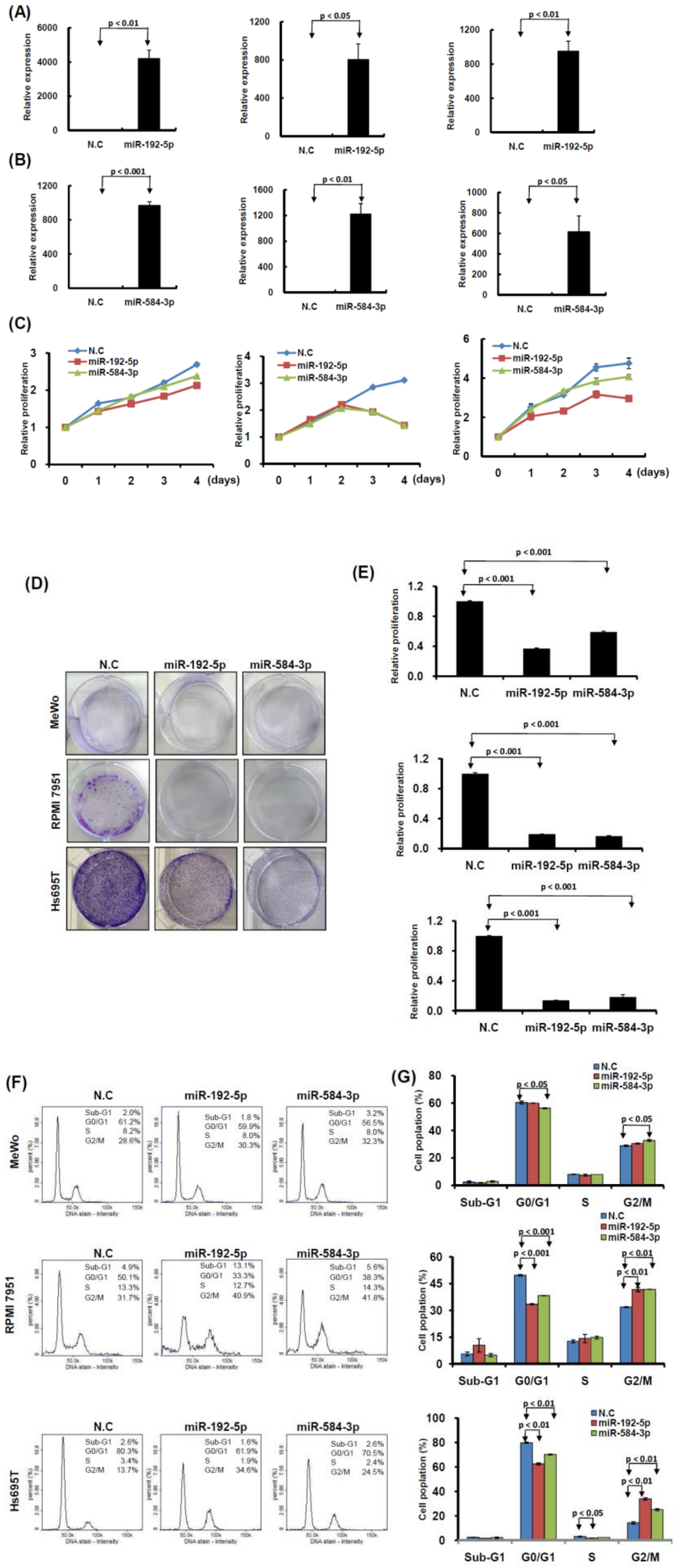
(A)
Relative expression
p < 0.01
p < 0.05
p < 0.01
N.C
miR-192-5p
(B)
p < 0.001
p < 0.01
p < 0.05
miR-584-3p
(C)
Relative proliferation
N.C
miR-192-5p
miR-584-3p
(days)
(D)
MeWo
RPMI 7951
Hs695T
(E)
p < 0.001
(F)
Sub-G1 2.0%
G0/G1 61.2%
S 8.2%
G2/M 28.6%
Sub-G1 1.8%
G0/G1 59.9%
S 8.0%
G2/M 30.3%
Sub-G1 3.2%
G0/G1 56.5%
S 8.0%
G2/M 32.3%
Sub-G1 4.9%
G0/G1 50.1%
S 13.3%
G2/M 31.7%
Sub-G1 13.1%
G0/G1 33.3%
S 12.7%
G2/M 40.9%
Sub-G1 5.6%
G0/G1 38.3%
S 14.3%
G2/M 41.8%
Sub-G1 2.6%
G0/G1 80.3%
S 3.4%
G2/M 13.7%
Sub-G1 1.6%
G0/G1 61.9%
S 1.9%
G2/M 34.6%
Sub-G1 2.6%
G0/G1 70.5%
S 2.4%
G2/M 24.5%
(G)
Cell poplation (%)
p < 0.05
p < 0.001
p < 0.01
Sub-G1
G0/G1
S
G2/M

Figure S2. Growth of melanoma cells was suppressed after transfection of miR-192-5p and miR-584-3p mimic candidates. (**A**) and (**B**) After transfection of miRNA mimics, the relative expression levels of miR-192-5p and miR-584-3p were examined in the MeWo, RPMI7951, and Hs695T cells through real-time PCR. (**C**) After miR-192-5p, miR-584-3p, and control, respectively, were transfected into the melanoma cells, cell proliferation was assessed. (**D**) and (**E**) Colony formation was examined with crystal violet and quantified using OD595 nm. (**F**) and (**G**) Cell cycle progression was examined and quantified in the MeWo, RPMI7951, and Hs695T cells after miR-192-5p or miR-584-3p transfection.

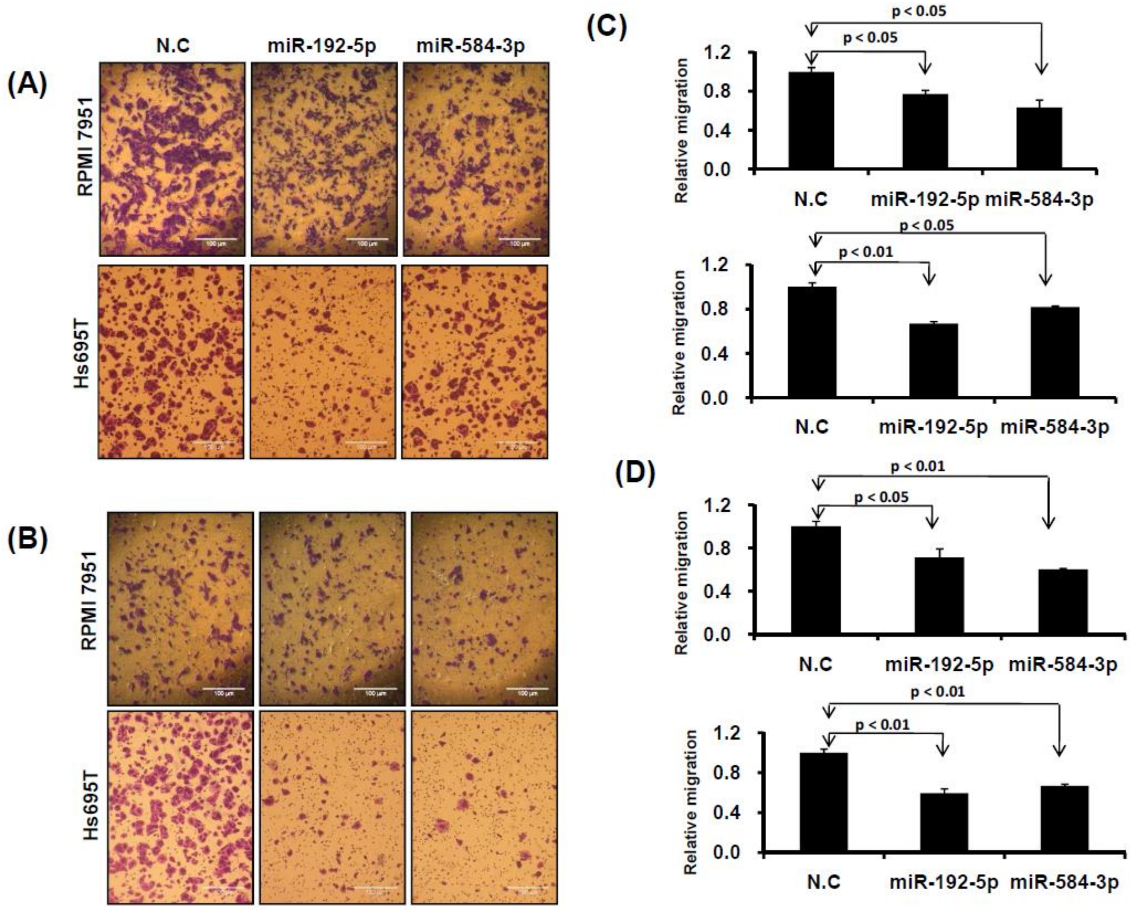

Figure S3. Motility of melanoma cells was suppressed after transfection with miR-192-5p and miR-584-3p mimic candidates. (**A**) and (**B**) The cell migration and invasion ability were examined in three melanoma cells, MeWo, RPMI7951, and Hs695T, after miR-192-5p or miR-584-3p transfection. (**C**) and (**D**) Then, the numbers of migrating or invading cells were quantified by counting three different fields under a phase-contrast microscope. The cell photographs from a representative experiment are presented, and the graph data were quantified using Ascent software. Data are reported as colonies compared with control (mean ± SD).

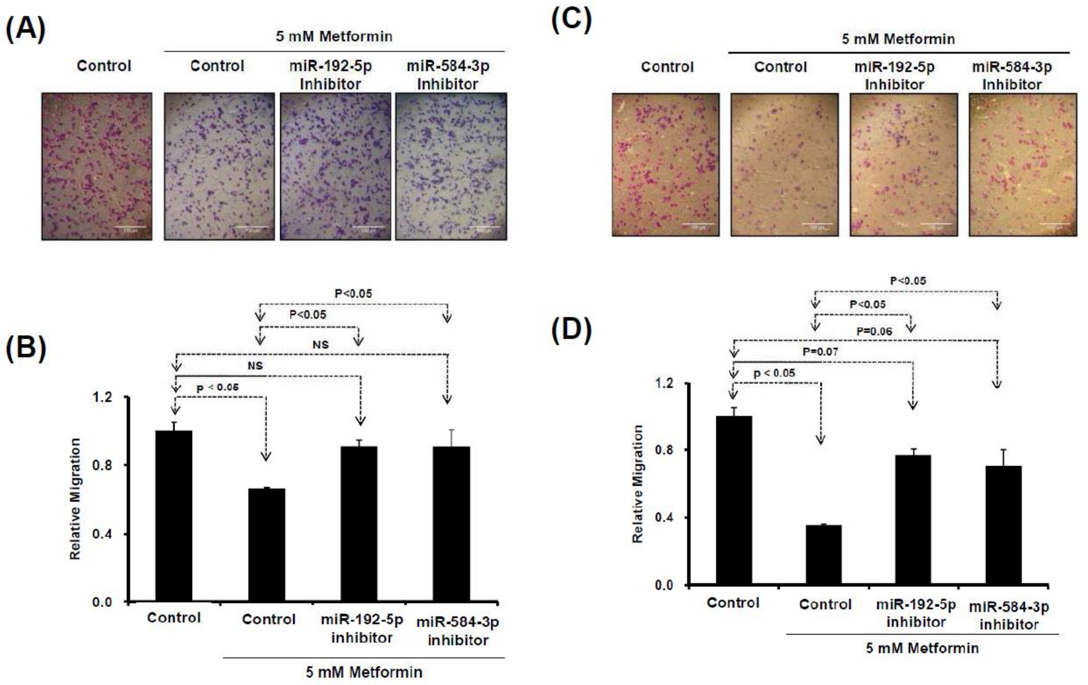

Figure S4. Partial improvement made by miR-192-5p and miR-584-3p inhibitors in the metformin-induced suppression of melanoma cell motility. (**A**) and (**C**) Migration ability was examined using a Transwell assay in A2058 and A375 cells with miR-192-5p and miR-584-3p inhibitor transfection. After transfection with the miR-192-5p inhibitor, miR-584-3p inhibitor, or scramble control for 24 h, the cells were treated with or without metformin and then subjected to a Transwell assay. Migrating cells were stained with a crystal violet solution. (**B**) and (**D**) Number of migrating cells was determined by counting three fields under a phase-contrast microscope. Photographs of the cells from a representative experiment are presented, and the graph data were quantified using Ascent software. Data are reported as colonies compared with the control (mean ± SD).

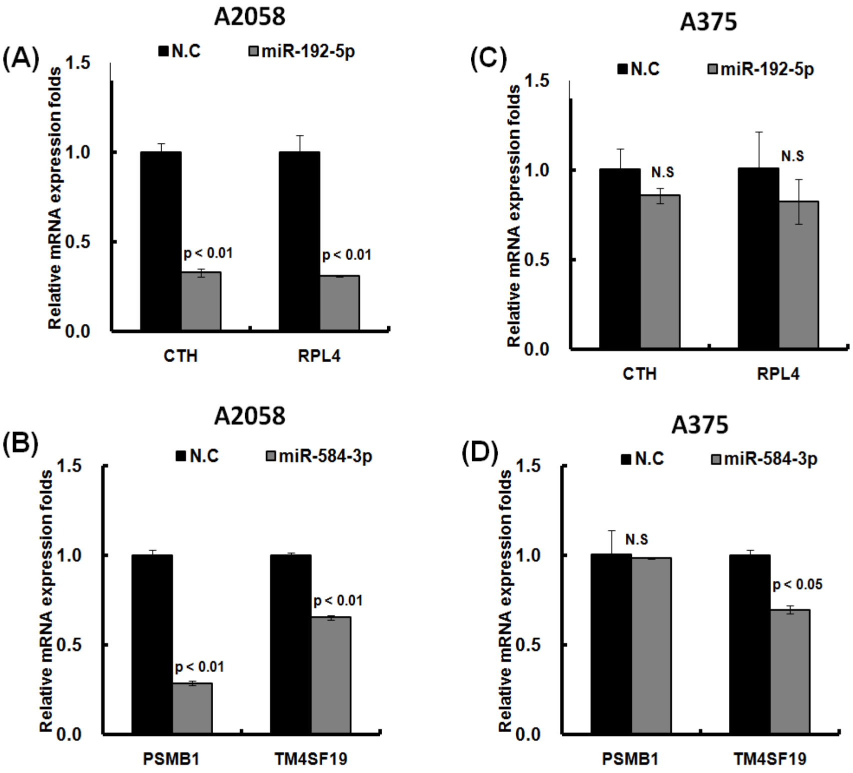

Figure S5. Expression levels of CTH, RPL4, PSMB1, and TM4SF19 were examined through real-time PCR in two melanoma cells with miR-192-5p (**A** and **B**) and miR-584-3p (**C** and **D**) transfection.

© 2019 by the authors. Licensee MDPI, Basel, Switzerland. This article is an open access article distributed under the terms and conditions of the Creative Commons Attribution (CC BY) license (http://creativecommons.org/licenses/by/4.0/).
